# A Comprehensive Review of Food Hydrogels: Principles, Formation Mechanisms, Microstructure, and Its Applications

**DOI:** 10.3390/gels9010001

**Published:** 2022-12-20

**Authors:** Pinku Chandra Nath, Shubhankar Debnath, Kandi Sridhar, Baskaran Stephen Inbaraj, Prakash Kumar Nayak, Minaxi Sharma

**Affiliations:** 1 Department of Bio Engineering, National Institute of Technology, Agartala 799046, India; 2 Department of Food Technology, Koneru Lakshmaiah Education Foundation Deemed to be University, Vaddeswaram 522502, India; 3 Department of Food Science, Fu Jen Catholic University, New Taipei City 24205, Taiwan; 4 Department of Food Engineering and Technology, Central Institute of Technology Kokrajhar, Kokrajhar 783370, India; 5 Haute Ecole Provinciale de Hainaut-Condorcet, 11, Rue de la Sucrerie, 7800 Ath, Belgium

**Keywords:** hydrogels, food applications, bioactive compounds, mechanical strength, microstructure

## Abstract

Food hydrogels are effective materials of great interest to scientists because they are safe and beneficial to the environment. Hydrogels are widely used in the food industry due to their three-dimensional crosslinked networks. They have also attracted a considerable amount of attention because they can be used in many different ways in the food industry, for example, as fat replacers, target delivery vehicles, encapsulating agents, etc. Gels—particularly proteins and polysaccharides—have attracted the attention of food scientists due to their excellent biocompatibility, biodegradability, nutritional properties, and edibility. Thus, this review is focused on the nutritional importance, microstructure, mechanical characteristics, and food hydrogel applications of gels. This review also focuses on the structural configuration of hydrogels, which implies future potential applications in the food industry. The findings of this review confirm the application of different plant- and animal-based polysaccharide and protein sources as gelling agents. Gel network structure is improved by incorporating polysaccharides for encapsulation of bioactive compounds. Different hydrogel-based formulations are widely used for the encapsulation of bioactive compounds, food texture perception, risk monitoring, and food packaging applications.

## 1. Introduction

In order to develop novel and innovative consumer-based functional foods, food processing industries are facing numerous challenges. Recent food processing trends are based on ensuring food safety and nutritional value to safeguard consumers. Furthermore, under proper storage, shipping, and delivery circumstances, the longevity of a product can be extended [1]. Researchers are creating new forms of food matrices that can be synthesized artificially [2] or with the assistance of different microbes [3], plants [4], or insects to assure quality and safety [5]. Foods with gelation characteristics have recently become much more popular in the marketplace due to their delicious taste, features that increase satiety, low calorie content, and high moisture content. These factors combine to make these foods healthier. In addition, rheological properties and sensory qualities of food hydrogels are essential for their excellent market price [6]. Natural gels made from food biopolymers have made significant strides over the past few decades, and in comparison, to their synthetic analogs, they have a wider range of applications and superior properties in a variety of contexts.

Gels are colloidal systems containing a continuous phase (i.e., a solid matrix) and a dispersed phase (i.e., aqueous solvent), leading to the formation of a semisolid texture [7]. The biopolymers that make up these gels are the primary ingredient that gives them their structure. Polysaccharides and proteins are the two types of biopolymers that are most frequently used. These biopolymers provide a variety of food products with a semisolid consistency. These biopolymer-based gels are composed of networks of various shapes, such as blocks, particles, or fibers, which exhibit a range of behaviors when subjected to mechanical stress [8].

Hydrogels are 3D polymer networks that are elastic because they are bound together by weaker bonding forces in the form of hydrogen or ionic bonds and crosslinked covalent bonds. These factors contribute to the ability of hydrogels to hold their shape. Hydrogels are used in current food design for a variety of purposes, including the creation of complex shapes through 3D printing, the replacement of fats, increased satiety with less food, and the maintenance of metastable structures of products [9]. Gels are also effective for reducing the unpleasant tastes induced by several bioactive compounds and medications because gel formation limits or slows molecular transport [10]. On the other hand, the use of flavor enhancers such as salt and sugar can be reduced by using liquid or brittle gels, which intensify the flavor [11]. If a gel is to be fully integrated into a liquid without altering the flavor of the food, its size must be drastically reduced before it can be used in liquid foods, such as beverages.

In order to satisfy the worldwide demand for food and provide sustenance for the growing human population, several food products that are environmentally friendly, inexpensive, safe, and packed with nutrients are required. In this context, food gels are considered sustainable food matrices containing a high concentration of nutrients. The employment of hydrogels mostly in the industry of food technology is not nearly as widespread as it is in other branches of the scientific community, such as tissue engineering and biomedicine. Therefore, the design of the structure of food hydrogels will require investigation of additional functional applications in order to advance the development of functional foods. Owing to the widespread sensorial acceptance of these medicinal and healthful foods, which were made possible by incorporating food gels, an increase in consumption of such food products has been observed [12]. When utilizing gels in food production systems, it is vital to consider how these substances will react inside the body. As a result, it is essential to take a number of factors into consideration in order to control the discharge or retainment of bioactive substances across the digestive system [13]. Enzymes and the acidic atmosphere of the stomach present the most significant challenge to maintaining the integrity of the shell for subsequent passages through the gastrointestinal tract because the stomach is the first organ that the shell must pass through [14]. Therefore, it is expected that the smart layout of the structure of the hydrogel will lead to an increase in the number of useful applications for new food items. In this review, we focused on the possible implementations and hydrogel interactions pertaining to the microstructure of various hydrogels and their food applications. The novelty of this review lies in the understating of the formation, rheological characteristics, and impact of the microstructure of hydrogels. An understanding of the interlaying mechanism of hydrogels could provide an opportunity for a wider range of applications in the food industry.

## 2. Gelling Agents

**Figure 1:**
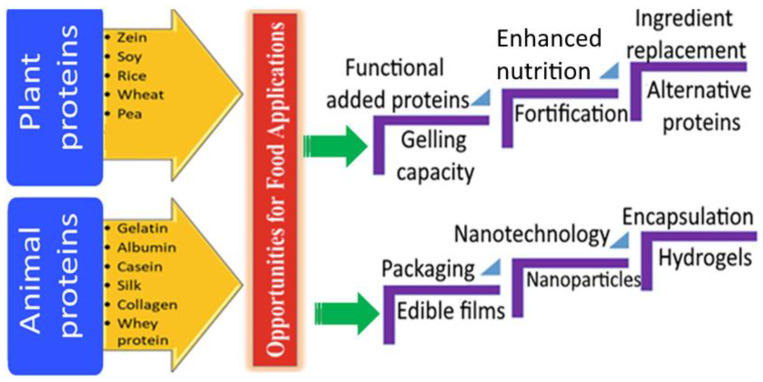
Different types of plant- and animal-based ingredients as gelling agents in the food industry. Figure 1 is reprinted with permission from Munir et al. [15] (Copyright © 2022 by authors).

Gelling agents are added to a wide range of foods, such as jellybeans, sweets, and chocolates, to make them thicker and more stable. As a result of gel formation, these ingredients give such foods their distinctive textures. In addition to their other functions, stabilizers and thickeners can also act as gelling agents. The application of plant- and animal-based proteins as gelling agents in the food industry is illustrated in Figure 1. Only a small fraction of gums can be used to make gels, and even among those that can be used for such purposes, there is a wide range of gel characteristics and textures that dictates their use in different food contexts. Natural gums, enzymes, pectins, starches, and agars are just some of the proteins and polysaccharides used as gelling agents. Heteropolysaccharides and hydrocolloids make up the vast majority of gelling polysaccharides (Table 1). Structures can be complex but well-defined repeating units, block copolymers, or either regularly or irregularly branched structures. They come from various sources, such as plant, animal, and microbial sources in nature. Gel formation is the primary focus, although thickening is a possible side effect. Jams, jellies, salad dressings, desserts, marmalade, jujubes, yogurts, etc., are just a few of the uses. However, many proteins are also employed in gel fabrication (Table 2). Corn zein and various animal proteins such as gelatin and whey are among these.

## 3. Conditions Necessary for the Formation of Gels

**Figure 2:**
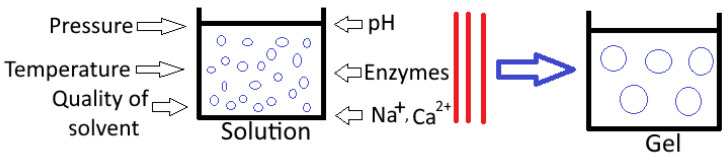
Different factors affecting the formation of food hydrogels.

Gel formation occurs as a consequence of the combination of a straightforward polymer dispersion or particle suspension with an extrinsically modifiable temperature or solution composition [16]. As a consequence of this, the process of converting sol-gels typically involves the aggregation of either particles or macromolecules, with the end result being the formation of a network that encompasses the entire volume of the container [15,17]. Gelation reactions can be roughly classified into two categories: those that are driven by physical forces (e.g., heat and pressure) and those that are induced by chemical processes (e.g., acidic, ionic, and enzymatic processes) [18,19]. The gelation of proteins requires a driving force to unfold the native protein structure, followed by an aggregation process, resulting in a three-dimensional organized network of aggregates or strands of molecules crosslinked by non-covalent bonds or, less frequently, by covalent bonds [20]. The numerous physicochemical characteristics covered in the subsequent sections are the primary determinants of the conditions that must exist for gel to form [21]. Figure 2 depicts the primary factors that influence the formation of gels and further information on each of these factors is addressed below in this section.

**Table 1:** Sources of gelling agents, as well as their textures, rheological properties, and applications.

Gelling Agent and Concentration	Source	Texture and Rheological Properties	Binding Blocks	Applications	Ref
Pectin (0.5–1%, *w*/*w*)	Heteropolysaccharides from higher terrestrial plant cell walls and fruits, such as citrus fruit, guava, and apple	Newtonian behavior	Partially esterified α-(1–4)-linked D-galactouronic and mannuronic acid; rhamnose, galactose, and arabinose can replace galacturonic acid	Jelly, jam, marmalade, fruit chews, and yogurt	[22]
Cellulose (0.5–2%, *w*/*w*)	Chemically modified plant cell walls	Shear thining crossmodelling and viscoelastic behavior	Homopolymer of D-glucose β-(1, 4)	Sweets and salad dressings	[22,23]
Agar (1–2%, *w*/*w*)	Red algae or seaweeds	Thermoreversible gels upon cooling; lowest viscosity at low shear rates; with increasing concentration of agarose, the particle size and particle surface decrease	Utilization of agarose and agaropectin together in a single product	Vegetarian gelatin and laxative	[22,24]
Guar gum (1–5%, *w*/*w*)	Endosperm	Although it is independent of electrolytes and has extremely high low-shear viscosity and high-shear thinning, it degrades and loses viscosity at high temperatures and low pH	Galactomannan molecular sequence	Sweets, yogurt, and various forms of liquid cheese, along with fillings for pastries	[25,26]
Carrageenan (0.5–3%, *w*/*w*)	Red seaweeds	High hardness, compressibility, adhesiveness, and cohesiveness	Sulfated D-galactose and L-anhydrogalactose	Desserts, cell/enzyme immobilization gel	[27,28]
Carob gum or locust bean gum (0.16–1.84%, *w*/*w*)	Carob tree seeds	High-shear thinning; very high low-shear viscosity, despite being degraded and losing viscosity regardless of the presence of electrolytes at high and low pH levels and high temperatures	Galactomannan	Glue	[29]
Xanthan gum (1–3%, *w*/*w*)	Xanthomonas campestris is responsible for the process of fermenting glucose, as well as sucrose	High-shear thinnability; maintains viscosity in the presence of electrolytes, high temperatures, and a wide pH range	Two -D-glucose units are linked at positions 1 and 4 along the polysaccharide chain; the primary structure has two mannose and one glucouronic acid, forming repeating modules of five sugar molecules	As emulsifiers, texture modifiers, fruit salads, and sauces, colloidal oil and solid ingredients are kept from turning into a cream	[30,31]
Alginate (1–2%, *w*/*w*)	Brown seaweeds	Pseudoplastic non-Newtonian and viscoelastic behavior	Different arrangements of (1-4)-linked -D-mannuronate and its C-5 epimer α-L-guluronate residues form a linear copolymer	Appetite-suppressant jellies, divalent ionic gelation, cell immobilization, and encapsulation	[32,33]
Gum Arabic (1–5%, *w*/*w*)	Acacia senegal and Acacia seyal sap	Low-viscosity gum; shear thinning occurs at low shear rates below 10/s, and near-Newtonian behavior occurs above shear rates of 100/s	Combination of saccharides and glycoproteins	Hard gummies, chocolates, and gums	[34,35]

**Table 2:** Application of proteins as gelling agents.

Gelling Agents	Source	Binding Blocks	Applications	Ref
Whey protein	Acid/sweet dairy whey	β-lactoglobulin and α-lactalbumin	Thickener and gelling agent	[36]
Soya proteins	Soybeans	Glycinin and β-conglycinin interact	Heat-set gel	[37]
Egg proteins	Egg	Albumen is made up of almost 70% globular proteins with ovomucin fibers and 30% egg white	Confectionery gelling and bulking agent	[38]
Zein	Corn	Peptide chain (prolamine)	Encapsulations in candies, nuts, fruit, and pills, along with other foods and baked products	[39]
Gelatin	Animal skin and bones	Glycine–proline protein	Jelly, jam, yogurt, and margarine	[40]

### 3.1. Pressure

Because high pressure may be used by itself or in conjunction with other processes, such as elevated temperatures, it provides more options for tailoring the functional molecular properties. Reactions that reduce the volume of a system are generally favored under high pressure. Dissociation of water at high pressure lowers its pH value. Gels formed under heat or pressure has distinct visual and rheological properties [41].

### 3.2. pH

The addition of acids or the fermentation of microorganisms can cause changes in pH, and these changes can alter the net charge of the molecule, which, in turn, can change the attractive and repulsive forces that exist between molecules, as well as the interactions among molecules and the solvent, also known as the hydration properties. In addition, the solubility of salts shifts depending on the pH, which is another factor that might play a role in gel formation. The theory of fractal aggregation may provide an explanation for the technique underlying the formation of acid gel [42].

### 3.3. Ionic Strength

A gel’s band gap can be increased by incorporating monovalent and divalent cations such as Na and Ca into the mixture. Gelation is possible as a result of the reduction or neutralization of the electrostatic repulsive forces that normally exist between molecules. It has been reported that pre-denatured whey proteins can undergo ionically induced gelation, which, in contrast to heat-induced gelation, is referred to as cold gelation [43,44]. In polysaccharide gels, such as xanthan gum, glucans, or guar gum, ionically induced gelation plays a more significant role than in other types of gels.

### 3.4. Temperature

The majority of gels are obtained by heating their constituents to a critical temperature [45]. The first step in the gelation process is uncovering or molecular fragmentation in response to the energy required, which exposes the reactive sites. Step two involves the joining or sticking together of unfolded compounds to make complexes with a higher molecular weight. Whereas the first process might be reversible; the second is almost always irreversible. Hydrophobic interactions and disulfide (-S-S-) bridges are likely to play essential roles. According to the relative reaction rates of the individual steps over a given temperature range, the unfolding or aggregation reaction can dictate the overall reaction rate.

### 3.5. Availability of Enzymes

Enzymes control complex procedures and active interaction in natural systems and their potential for building self-regulating soft matter systems is growing [46]. Enzymatically induced gelation works by adding artificial covalent cross links to food proteins. Protein crosslinking works best with reactions set off by transglutaminase (TG), polyphenol oxidase, and lipoxygenase [47].

### 3.6. Gelling Agent Concentration

Gel formation is only possible once the critical minimum concentration (denoted by the symbol C^∗^), which is unique to each hydrocolloid, has been reached. Agarose can form gels at concentrations as low as 0.2%, whereas acid-thinned starch requires a concentration of at least 15% before gels can be formed [48].

### 3.7. Solvent Quality

The nature and presence of solvent has a significant effect on gel formation; for instance, concentrated sugar solution is an ineffective solvent for pectin [47]. Only in a concentrated sugar solution can hydrogen bonds form in the junction zones, so gelation can only occur in this type of solvent [49].

## 4. Gel Types and Mechanism of Gel Formation

Food gels can be categorized in various ways based on their biopolymer networks, gelation mechanisms, morphologies, interactions, etc. Gel structures can be classified either as simple networks (made up of a single biopolymer such as polysaccharides or proteins) binary/mixed networks (made up of two or more biopolymer types), or composite/filled networks (made up of a biopolymer network and various other particles such as fat globules) [18]. Cold-set gels, heat-set gels, ionotropic gels, acid or enzymatically induced gels, etc., are all distinct types of gels that form for different reasons. Gels can be classified as either filamentous or crystalline or as a soft particle suspension, depending on their morphological characteristics.

Gels may contain a variety of interactions, including hydrogen bonding, interactions involving hydrophobic groups, non-covalent interactions, and covalent chemical bonds. Among the many types of chemical bonds, H-bonding is the most common type of interaction. Polymer gels can be divided into three categories: strong, weak, and pseudogels, all of which are fundamentally determined by the three-dimensional layout of the biopolymer network [7]. Hybrid gels of polymers that have been subjected to chemical crosslinking are regarded as particularly robust gels. The crosslinks in these gels are irreversible and cannot be repaired if they become broken. Colloidal gels and certain biopolymer gels are examples of weak gels that contain crosslinks that are capable of being broken and reformed [50,51]. Long-lasting physical interactions within the polymer matrix can function like chemical crosslinks, which gives these materials gel-like properties. Therefore, polymer systems that get tangled up are sometimes called “pseudogels” [51]. If the stress is kept constant, the steady-state response of a pseudogel to an extreme stress is to flow like a fluid. It is possible that different criteria could be used to classify food gels; however, in order to make it simpler to comprehend the methodology of gelation and the microstructure of gels, in this chapter, we describe gels based on the category of the biopolymer system they contain.

As previously stated, gelling agents include any of the polysaccharides and proteins found in plants, animals, and microbes [52]. Polysaccharides, also known as natural gums, are used frequently in the food industry and include ingredients such as agar, guar gum, alginate, xanthan gum, starches, and glucans. The most popular proteins contain pectin, glutenin, skim milk, soy milk, egg albumin, and zein. Many of these proteins and polysaccharides are employed as thickeners in culinary creations. The viscosity and shear modulus can differentiate between thickening and gelling. Gel formers usually increase the solubility until the gel point, when the viscosity becomes (possibly) infinite and the elastic modulus above the gel point is finite [53]. The first steps in the gelling process are the uniform dispersion of the gelling agent, followed by the hydration of the agent. The subsequent formation of the network is responsible for giving the product its texture.

### 4.1. Polysaccharide Gels

**Figure 3:**
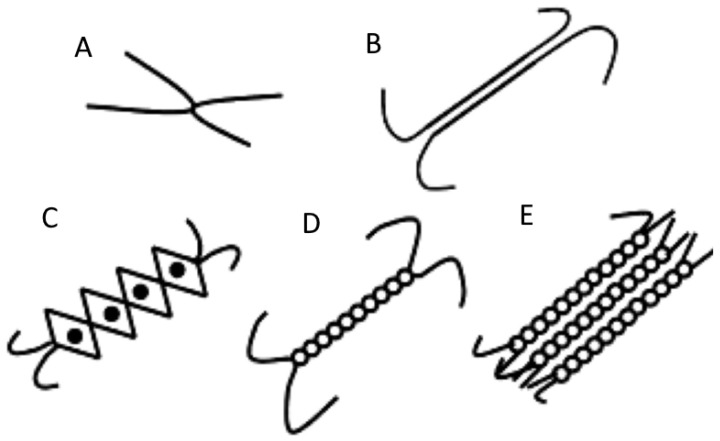
Different junction zones in polysaccharide gels: (**A**) connecting points, (**B**) extended junction zone resembling a block, (**C**) model of crosslinks in alginate and pectin gels based on an egg-box structure, (**D**) the region of the double-helix junction, and (**E**) junction zone for the aggregation of polysaccharide chains. Figure 3 is reprinted with permission from Nazir et al. [8] (Copyright © 2017 Elsevier Ltd., Amsterdam, The Netherlands).

There are just few polysaccharides that can form a gel when exposed to a certain concentration of the gelling agent, which is typically referred to as the critical concentration. Other polysaccharides, on the other hand, are utilized in various foods as thickeners and stabilizers. Polysaccharide critical concentrations are significantly lower than those of protein critical concentrations. After polysaccharides have been completely hydrated, the polymer strands begin interacting (crosslinking) with one another in order to produce junction zones. When the polymer solution (dispersion) reaches a certain critical polymer concentration and a certain degree of crosslinking, the polymer solution eventually transforms into a gel that possesses a stable network structure. The composition of the polysaccharide and the conditions under which the gel is formed can result in a wide variety of junction zone configurations (Figure 3). Some polysaccharides, known as thermos or heat-set gels, crystallize when heated and then cooled again, whereas other polysaccharides, known as cold-set gels, do so at room temperature by utilizing particular types of cations, adjusting the pH of the solution, or incorporating particular cosolutes. Curdlan is a special polysaccharide because it can produce gels that are heat-set as well as cold-set. This ability sets it apart from other polysaccharides [54]. In the following sections, we discuss polysaccharide gels, which are utilized the vast majority of the time [55].

#### 4.1.1. Alginate

In the presence of divalent cations, alginate gels are produced, and the strength of the resulting gels is directly proportional to the type of cation used. Ca^++^-induced gelation is the most significant among the various cation-induced gelations for functional foods [56]. In general, alginate gels are not affected by heat and are irreversible. Egg-box structures are formed when cations begin a linkage among polyglucuronic acid regions of adjacent polymers. This linkage occurs because buckled conformations in the polymers provide efficient binding sites. Egg-box structures are also known as egg crates. Because alginates can form a gel even when the temperature is just above room temperature, they can be used in a variety of food applications.

#### 4.1.2. Pectin

The degree of esterification of pectin significantly impacts the gelling properties exhibited by pectin [57]. Pectin’s with a high methoxyl content only set into a gel when sugars or other cosolutes are present [58], such as carbohydrates, polyols, or monohydric alcohols, and with a sufficiently low acid concentration (3.0 to 4.5) [59]. Low-methoxyl pectins are capable to formgel in the presence of divalent compounds, such as calcium.

#### 4.1.3. Agar

In both bacterial media and food products, agar gels experience a synergistic effect [60]. Agarose, a neutral polysaccharide, and agaropectin, a charged polysaccharide with sulfate groups, combine to form an agar gel. Accordingly, the gel osmotic pressure increases, and the degree of syneresis decreases as the agaropectin fraction increases [61]. In most cases, the total sulfate content has an inverse proportional relationship with the amount of water extracted from the agar gel. The total polymer concentration in the gel follows the same pattern. Intriguingly, the extent of syneresis in agar gel was found to be roughly inversely proportional to the square of the concentration. The uncharged polymer’s osmotic pressure scales approximately to this value.

#### 4.1.4. Starch

**Figure 4:**
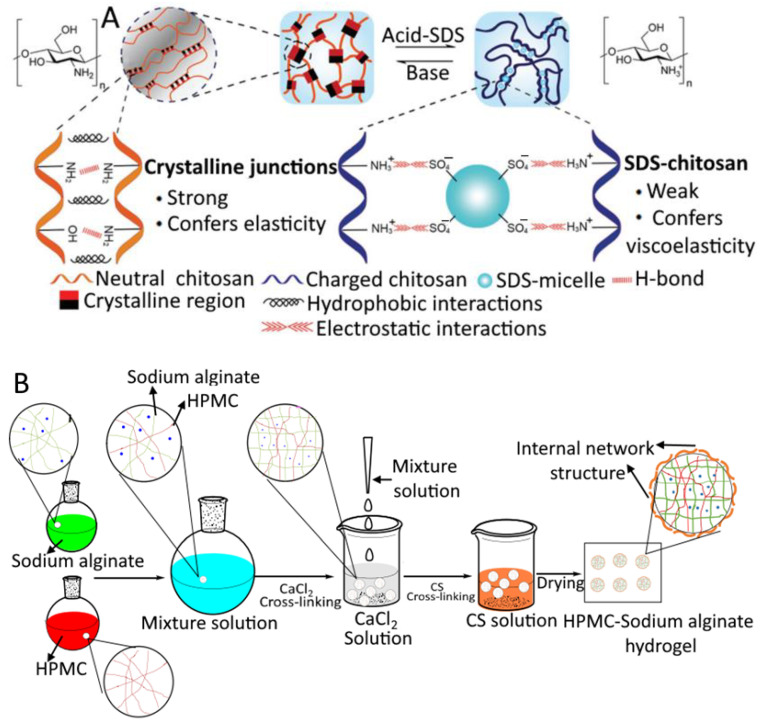

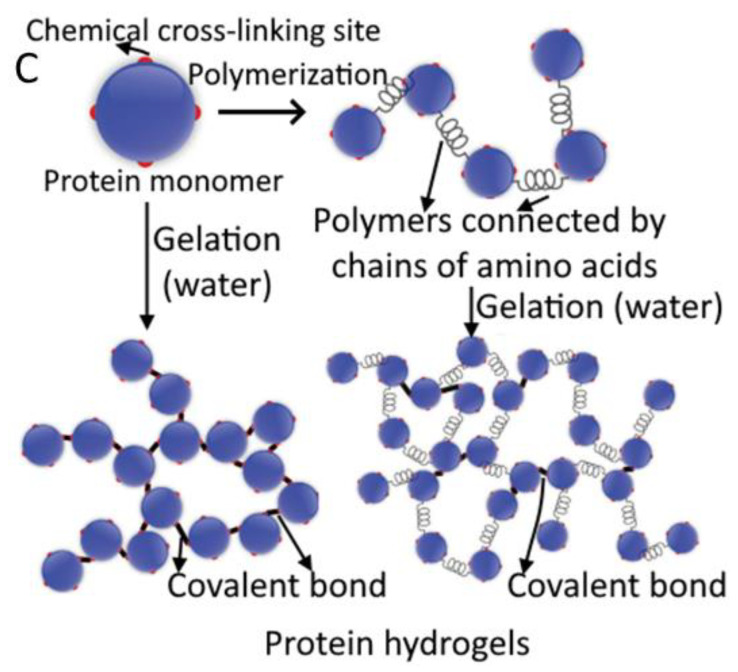
Mechanism of polysaccharide and protein gel formation. Crosslinking of chitosan (**A**); composite hydroxypropyl methyl cellulose-sodium alginate hydrogels (**B**); gelation process of chemically crosslinked protein hydrogels (**C**). (**A**,**B**) are reprinted with permission from He et al. [89] (Copyright © 2017 Wiley-VCH Verlag GmbH & Co. KGaA, Weinheim, Germany) and Hu et al. [90] (Copyright © 2018 Springer Nature B.V., Dordrecht, The Netherlands), respectively, whereas (**C**) is reprinted from Hanson et al. [91] and is an open-access article (Copyright © 2020 by authors) distributed under the terms and conditions of the Creative Commons Attribution (CC BY) license. HPMC, hydroxypropyl methyl cellulose; CaCl_2_, calcium chloride; CS, chitosan.

Amylose, a linear chain, and amylopectin, which is branched, are the two types of polymers that make up starch granules (branched) [62]. Granules are embedded within an amylose matrix in the composite gel that is formed as a result of its formation. A process known as gelatinization is responsible for the gelation of starch. During this process, starch granules are heated, which causes them to absorb a sufficient amount of liquid and swell to several times their original size. After being cooled, the amylose fraction becomes ordered (in the form of single helices) around the swollen granules and is leached out by the granules. The method that causes the starch to gel differs depending on the source of the starch; for example, chitosan-based hydrogel and its mechanism are illustrated in Figure 4A.

#### 4.1.5. Carrageenan

Carrageenan is a type of ionic polymer that, when cooled in the presence of salts (electrolytes), particularly potassium ions, forms helical gels. Both the formation of helices and the setting of gels can be facilitated by particular cations, such as K^+^, Rb^+^, Cs^+^, and NH_4_^+^. A coil–helix transition is experienced by molecules, followed by the aggregation of helices [63]. The formation of “ordered domains” is a common step in carrageenan gelation [64], which typically involves the association of polymer chains through the formation of intermolecular double helices. Gelation takes place after the subsequent aggregation of these domains, which is mediated by the specific binding of the cations that are responsible for gelation. The transformation of carrageenan from its disordered, random coil state into its ordered, helical state is the first step in the gelation process [65].

#### 4.1.6. Gellan Gum

In terms of their chemical composition, gellan gums can be broken down into two categories: high- and low-acyl gellan gums [66]. Gels with high-acyl gellan tend to be soft and elastic, whereas gels with low-acyl gellan tend to be hard and brittle [67]. The presence of ions facilitates the association of double helices, which, in turn, leads to gelation.

#### 4.1.7. Cellulose Derivative Gels

##### Carboxymethyl Cellulose (CMC)

CMC is a water-soluble cellulose derivative widely used in the biopolymer industry. It is produced by replacing 2, 3, and 6 hydroxyls on the backbone of cellulose with carboxymethyl groups [68]. Cellulose containing numerous hydroxyl groups is an abundant and inexpensive natural biopolymer, making it a desirable starting material. CMC also exhibits bioactivity, solubility, and biodegradability. CMC is prepared in a nonaqueous monochloroacetic acid/soda solvent medium to achieve the substitution degree via carboxymethylation [69].

CMC-based hydrogel has the potential for use in enzyme immobilization [70], wound healing [71], drug delivery [72], and adsorption [73]. Hydrogels from nanoparticles/CMC can be used for their antimicrobial properties [74], wound healing, drug development, and tissue engineering [75]. The performance of carboxymethyl cellulose hydrogel is enhanced by the addition of nanoparticles [76]. Nanoparticles enhance carboxymethyl cellulose hydrogels by virtue of their superior mechanical, electronic, optical, and physicochemical properties. Carboxymethyl cellulose derived from pineapple plants is an effective vehicle for papain immobilization and forms a strong hydrogen bond between the employed materials [77]. Although CMC can be easily extracted from biomass resources [78], bagasse [79] and empty fruit bunch [80] have also been used to produce carboxymethyl cellulose. Every type of biomass resource imparts unique characteristics to CMC, such as exceptional absorption and adsorption, a high swelling ability, and superior optical properties. In addition to being advantageous for the production of CMC hydrogels, a high level of methylation groups in various types of biomass waste is also beneficial [81].

##### Hydroxypropyl Methyl Cellulose (HPMC)

Cellulose derivative HPMC is utilized extensively in controlled-release applications, owing to its ability to thicken, gel, and swell. It is also safe to use, easy to compress, has properties that make it swell, and can handle high drug levels.

Owing to its excellent bioactivity, HPMC can be a thermosensitive natural polymer that forms a transparent, highly stable, colorless hydrogel, with positive rheological properties and changes in the texture. Gårdebjer et al. [82] investigated the pore-forming effects of hydroxypropyl methylcellulose, mostly in MFC (micro fibrillated cellulose) film, and made adjustments to the wettability characteristics of the films. The results demonstrate that HPMC can potently react with MFC films, possibly creating H-bonds on the surface of the film.

Hydroxypropyl methylcellulose for use in scaffold engineering was created from crosslinked chitosan by Boyer et al. [83]. After promoting cellular characteristics, they demonstrated that crosslinking hydroxypropyl methylcellulose with chitosan can endow the recovery process with structural strength and shape. The use of HPMC as a composite hydrogel in scaffold engineering was also investigated by Bacakova et al. [84]. Likewise, HPMC is widely used to make composite hydrogels using different polysaccharides (Figure 4B). Overall, HPMC composite hydrogel can facilitate faster healing, a more uniform distribution of cells, and a reduced risk of complications during osteoplasty procedures. Hydrogel scaffolds, films, and membranes are some of the typical applications for HPMC in the sector of medicine.

##### Hydroxypropyl Cellulose (HPC)

HPC can be dissolved in ice-cold water, and its viscosity changes with pressure. Temperatures higher than 45 °C render HPC insoluble, and unlike MC and HPMC, it does not form a gel. HPC, on the other hand, may be soluble in ethanol and ethanol/water mixtures. A reversible precipitation process begins when the polymer reaches a temperature above 45 °C [85]. However, sucrose or high concentrations of salt can be introduced to lower the deposition temperature [86]. When heated, viscosity drops dramatically—typically by half for every 15 °C [87]. HPC maintains its viscosity over a wide range of pH values, from 2 to 11. However, solutions should be stored at a pH of 6–8 to prevent the loss of viscosity due to decomposition [87].

### 4.2. Protein Gels

Generally, proteins are the convenient biopolymers for the formation of food-based hydrogels. Moreover, plant and animal-based proteins are widely used in the fabrication of food-based hydrogels. The mecahnisms of protein-based hydrogels are illustarted in Figure 4C.

#### 4.2.1. Gelatin

It is appropriate to begin the discussion with gelatin gel-containing food products, for which the term syneresis was coined. It has been reported that gelatin gels with a pH below or above the isoelectric point swell considerably [88]. These gels are stable at these pH levels and exhibit no syneresis, even at low protein concentrations. The osmotic pressure resulting primarily from the Donnan equilibrium is responsible for the swelling when the gel is close to its isoelectric point of 4.7; however, different results have been observed. Below a 10% concentration, the gel deswells (syneresis), whereas above this threshold, it swells. Osmotic pressure responds more quickly to changes in concentration than the network pressure, comparable to the behavior observed in a chemically crosslinked, uncharged gel. It is abundantly clear that the osmotic pressure of gelatin gel appears to perform the most significant part in the process of triggering syneresis. Increasing the polymer concentration boosts osmotic pressure, which helps to prevent syneresis. It is also possible to increase osmotic pressure by shifting the pH away from the isoelectric point, which causes the polymer charge to increase.

#### 4.2.2. Whey Proteins

Whey proteins undergo a series of transitions that are characteristic of globular proteins before and during heat-induced gelation. These transitions include (i) the inactivation of native proteins, also known as their unfolding; (ii) the agglomeration of unfolded compounds; (iii) the formation of strands from aggregates; and (iv) the association of strands into a network. The presence of salts allows for the formation of aggregates to take place [88,92,93].

#### 4.2.3. Egg Albumin

Egg albumin is often thought of as a system made up of many different globular proteins dissolved in water [94]. The formation of egg albumin gel occurs in three steps. First, heating causes molecules to partially unfold; this results in an increase in the number of interactions between molecules. In the second stage, sulfhydryl disulfide exchange ultimately leads to molecular aggregation, and sulfhydryl oxidation occurs both within and between the network-forming aggregates. Multiple hydrogen bonds are generated in the final step of the process, which involves cooling. The process of gel formation can be affected by a number of factors, such as the pH of the solution, the presence of salts or sugars, and the ionic strength.

#### 4.2.4. Soy Proteins

Although acidification (for example, glucono-δ-lactone) can also induce aggregation of denatured protein molecules, gelation can be achieved by heating soybean flour [37] or milk, followed by the addition of salt (for example, Ca^++^ or Mg^++^), to form a gel or curd.

#### 4.2.5. Milk Proteins

Owing to the high hydrophobicity of casein molecules, submicelles of casein are held together through the formation of hydrophobic bonds and salt bridges. Rennet gelation occurs as a consequence of the enzymatic hydrolysis of k-casein by rennet, which results in the release of CMP (caseinomacropeptide) and the aggregation of micelles [47,95,96].

## 5. Rheological Characterization of Gels

Rheological characterization is important in determining a gel’s composition, structure, and the impact of processing on that structure. In addition, by adjusting the polymer microstructure and surrounding media, a gel with the required characteristics can be created. Modern rheometers can precisely measure the reaction of a complicated material to applied stress or strain. However, understanding the gelling process and manipulating the gel’s microstructure with desired sensory properties require a basic knowledge of rheology [97]. The crosslinked polymer networks in gels are what give them their viscoelastic properties. The type and degree of crosslinking in a polymer affect its strength, which is determined by equilibrium modulus, a rheological parameter. The polymer’s mean molecular weight increases prior to the process of crosslinking, which ultimately causes the polymer to transform from a liquid (sol) into a solid (gel); this transition point is termed the gel point. There are various rheological methods for determining whether a polymer is a gel. In a creep test, the material is subjected to a constant stress, and the rate of deformation is recorded. A gel is a viscoelastic material; thus, it has two components (elastic and viscous), which produce a characteristic increase in the creep compliance (*J*(*t*) = γ(t)/σ_0_, where γ(t) is the time-dependent shear strain, and σ_0_ is the constant stress applied to the sample in the creep step). Creep-recovery compliance is a measurement of the network elasticity of gels [98]. Therefore, it is difficult to achieve a constant deformation in any gel network. Gels differ from liquids and tend to dissolve in solvents, which causes them to swell. A gel’s equilibrium modulus affects how much it swells, with harder gels swelling less than softer gels. The most accurate way to determine the critical gel point—whereby a small amount of oscillatory strain (or stress) is applied to a material to see how it reacts—is through linear oscillatory rheology. Owing to the viscoelastic properties of gels, two rheological parameters can be used to describe their viscoelasticity: viscous or loss modulus ($G^{\prime \prime }\propto \cos\;\left(\omega t\right)$) and elastic or storage modulus ($G^{\prime }\propto \sin\;\left(\omega t\right)$). Elastic characters prevail when *G*′ is greater than *G*″, and viscous characters prevail when *G*′ is smaller than *G*″. Mechanical spectra can be expressed in terms of the complex modulus (G*) [99].

(1) $$G^{*}=\left[\sqrt{G^{\prime \prime 2}+G^{\prime 2}}\right]$$

(2) $$G^{*}=\left[A_{n}\omega^{n^{*}}\right]$$

where *A_n_* is the gel strength of samples, serving another characteristic parameter of the material structural type; *n*^∗^ is the power law exponent, serving as an index of the viscoelastic nature of the material (a measure of physical crosslinks in the protein network); and ω is angular frequency [99].

The viscoelastic moduli provide a measurement of the shear-deformation resistance in terms of the elastic-deformation resistance (*G*′) and the viscous-deformation resistance (*G*″). Both moduli are the relation between shear stress (force/unit of area (Pa)) and shear strain. They frequently have a significant amount of influence due to the period of distortion. The relative importance of the elastic modulus (*G*′) and the viscous modulus (*G*″) is reflected by the phase angle, which is calculated as tanδ = *G*″/*G*′, where δ is the phase angle. The loss factor (tanδ) provides a measurement of the viscoelastic degree of gels, together with the energy cohesiveness of samples, as it is related to the lifetime of bonds, which conform the polymeric network [100]. The storage modulus (*G*′) surpassing the loss modulus (*G*″) at a particular frequency, is among the criteria that determine whether a solution will turn into a gel.

### 5.1. Microrheology of Gels

Despite being macroscopic parameters, the viscoelastic data of gels can provide structural information, as they are fundamental data. Therefore, fundamental tests (oscillatory tests, creep and recovery tests, dynamical thermo mechanical analysis, etc.) are essentially independent of the measurement conditions, reflecting true material properties. These material properties are related to the structural characteristics [101,102]. Recent technological advancements have made available new strategies for comprehending complicated gel structures and dynamics with different characteristic and timeframes. As a result, the concept of microrheology has been introduced, which concerns how materials store and release mechanical energy as a function of length scale. By connecting the microstructure of food systems to their macroscopic characteristics and stability, such knowledge aids food scientists in understanding the fundamental mechanisms that influence the interactions of food components [103].

**Figure 5:**
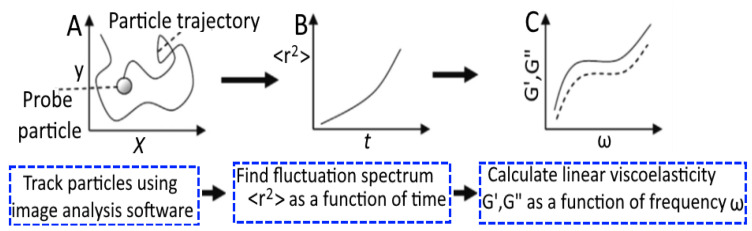
The linear viscoelasticity of low-modulus materials extracted from the fluctuation spectrum using particle-tracking microrheology. (**A**) The probe particle’s trajectory is calculated; (**B**) the spectrum of average fluctuations is computed as a function of time (t′); and (**C**) mechanical spectrum in the linear viscoelastic region (LVER). Figure 5 is reprinted with permission from Nazir et al. [8] (Copyright © 2017 Elsevier Ltd., Amsterdam, The Netherlands).

Particle-tracking rheology, magnetic tweezers, diffusing-wave spectroscopy, atomic force microscopy, laser particle tracking (optical tweezers), quasi-elastic light scattering and piezorheometery are just a few of the methods that can be used to characterize microrheology. Particle-tracking microrheology, also known as video particle-tracking microrheology, is one such method beginning to gain popularity. It has been used to characterize a variety of food gel systems. The local dynamics of soft material are examined in this scenario using the Brownian motion of embedded particles or tracers, without the application of an external driving force. As shown in Figure 5, there are three steps required to calculating linear viscoelasticity using particle tracking. Following tracking of particle trajectories and calculation of the mean-square displacement (MSD) as a function of the lag time of these fluctuations (due to Brownian motion), the data are translated into viscoelastic properties, specifically storage (*G*′) and loss (*G*″) moduli and creep compliance (J) [104]. With the least possible disruption to the developing gel structure, it can also identify the gel point.

The time-averaged mean-squared displacement in the picture plane (*x*, *y*) ({$∆r^{2}\left(\tau \right)\}$) is expressed in Equation (3):

(3) $$\left\{∆r^{2}\left(\tau \right)={\left[x+\left(t+\tau \right)-x\left(t\right)\right]}^{2}+{\left[y+\left(t+\tau \right)-y\left(t\right)\right]}^{2}\right\}$$

The calculated $∆r^{2}\left(\tau \right)$ corresponds to:
$∆r^{2}\left(\tau \right)=2dD\tau =2d\left(\frac{k_{B}T}{6\pi \eta a}\right)\tau$for viscous medium$∆r^{2}\left(\tau \right)=dk_{B}T/3\pi aG^{\prime }$for an elastic medium
where *D* is the diffusion coefficient calculated according to the Stokes–Einstein Equation (4).

(4) $$D=\left(\frac{k_{B}T}{6\pi \eta a}\right)$$

where *k_B_* is the Boltzmann constant. The calculated $∆r^{2}\left(\tau \right)$ is converted into elastic and viscous moduli. Additionally, the estimated $∆r^{2}\left(\tau \right)$ can be connected to another significant viscoelastic parameter, i.e., creep compliance (J(t)), which is expressed as the time-dependent strain that results from stress values within the linear viscoelastic range (LVER) [98].

### 5.2. Oral Processing and Texture Perception of Gels

Oral processing refers to the manipulation of food in the mouth, which produces signals transmitted to the brain and result in an impression of texture and mouth feel [11]. Several technical characteristics, such as cutting, elasticity, firmness, effort, extensibility, melting, and the adhesiveness rate in the mouth, provide a more nuanced explanation of a food’s texture. Texture is not just how food feels in the mouth, such as smooth or rough, light or heavy, etc. Given that gels are colloidal dispersions, which are typically thick, sticky, and solid-like materials, and thus, *G*′ > *G*″ → tanδ < 1 (elastic nature), as supported by the continuous phase (solid matrix), consumers may have trouble swallowing them, particularly the elderly and those who have dysphagia. Mastication (the act of chewing food) requires a variety of muscles, including the masseter muscles.

Suprahyoid muscles play a role in the movement of the tongue against the hard palate and the first movement of food during oropharyngeal swallowing. When eating soft foods such as jellies, suprahyoid muscles are more active than masseters; in contrast, eating firm, viscous, and sticky foods or gels causes more masseter activity than suprahyoid activity [105]. Electromyography (EMG), a method for evaluating the participation of masticatory muscles needed for chewing and swallowing various types of food, can be used to measure the activity of these muscles. EMG investigations are therefore helpful in research on food texture [106]. In one study, five different kinds of hydrocolloid gels were chosen, and EMG tests were run utilizing a variety of variables, such as the number of chews, the length of each chew, the quantity of masseter or suprahyoid action, the length of each masseter or suprahyoid action, etc. The findings indicate that chewing requires less effort with gels that melted more readily in the mouth than with hard and slick gels [107]. The fact that all types of gels exhibit strong masseter activity and have high EMG variable values can be explained by the fact that gels are always chewed thoroughly before ingesting.

## 6. Network Structure and Strength

### 6.1. Interconnected Polymeric Networks

Generally, the mechanical strength of typical food gels is sufficient for three-dimensional printing and edible film production. However, the networking of gels should be improved for encapsulation application [108]. In this regard, loading capacity is also limited for successful encapsulation of bioactive substances. Owing to the limitations of ordinary hydrogels, interpenetrating network hydrogels with superior mechanical characteristics have been developed. Interpenetrating network hydrogels are polymers composed of at least two networks that are partially intertwined on a molecular scale but not covalently bound to one another. Edible interpenetrating hydrogels are often made with natural polysaccharides and proteins, which are biodegradable and biocompatible. Several methods for producing interpenetrating hydrogels have been devised, including ionic [109], enzymatic [110], and heating and cooling [111] processes. Previously, various researchers developed hydrogels for application in food compositions to improve mechanical characteristics [112,113].

### 6.2. Strengthening Polymeric Networks

Enhancing the adhesion between distinct gel networks can boost the mechanical strength of hydrogels [114]. In previous research, two primary mechanisms have been implicated in an increase in the strengthening mechanism: polysaccharides incorporated as fillers of the network developed by other biopolymers, such as polysaccharide–protein hydrogels, e.g., k-carrageenan/sodium alginate double networks [115]; a myofibrillar protein embedded with native starch, such as (tapioca or potato starch) [116]; tilapia myofibrillar protein embedded with konjac glucomannan [117]; and myofibrillar protein imbedded in cassava starch [111]. Polysaccharides incorporated in proteins produce a hydrogel with an improved textural profile, possibly as a result of the “filling effect” or “packing effect”. Starch particles compactly linked to proteins in these hydrogels exert a “packing effect” by expanding the starch granules and increasing the ensuing internal pressure. As a result, enhanced strengthening of textural and mechanical qualities is observed [118]. Furthermore, other polysaccharides, such carrageenan and konjac glucomannan, primarily operate as fillers to improve the gelling properties of protein gels after hydration, a phenomenon known as the “filling effect”. These polysaccharides, when employed as fillers, can improve the mechanical properties of hydrogels by forming a dense and continuous gel network, enhancing the hydrogels’ adhesiveness, hardness, and chewiness [119]. Polysaccharides are subsequently interwoven with other biopolymers to improve the density of entangled networks, which are commonly found in polysaccharide–polysaccharide hydrogels [120]. In one investigation, hydrogels made of konjac glucomannan and flaxseed gum were made by heating and cooling the mixture. As the mixture cooled, hydrogen bonds formed between the flaxseed and konjac glucomannan molecules. Hydrogen bonds created strong interactions between molecules that worked together to make the hydrogel hard, springy, and chewy [49].

## 7. Application of Hydrogel-Based Formulations in the Food Industry

### 7.1. Fat Replacers

An increase in the consumption of high-fat foods is linked to an increase in the prevalence of chronic diseases, such as heart disease, obesity, and diabetes. As a result, it is essential to develop food products that contain less fat, such as mayonnaise and ice cream. On the other hand, reducing the amount of fat in foods can result in unfavorable changes in the finished product, such as reduced water retention, altered flavor, and a more rigid consistency. For instance, when fat is removed from cakes, the crumb mixture becomes firm and dry. In addition, the functional qualities of salad dressings may suffer as a result of reduced fat content [30]. Owing to their high capacity to hold water, hydrogels made from protein and polysaccharides have proven to useful in maintaining lubricity and texture in a manner comparable to that of full-fat products [119].

While using hydrogels to replace fat, it is preferable to have hydrogels with excellent viscoelasticity and rheological properties. Therefore, various hybrid hydrogels were developed for desirable characteristics, such as pectin and whey protein [119]. Researchers produced mayonnaises with pectin and whey protein hydrogel by replacing 20% of the fat contents. Furthermore, the addition of fat replacers at sixty percent concentration can improve the stability of mayonnaise by reducing the level of flocculation and coalescence of fat droplets [121]. In addition, a number of different biopolymers, such as starch, have been reported for use as fat replacers [122].

### 7.2. Encapsulation of Bioactive Compounds: Gel-Based Formulations

**Figure 6:**
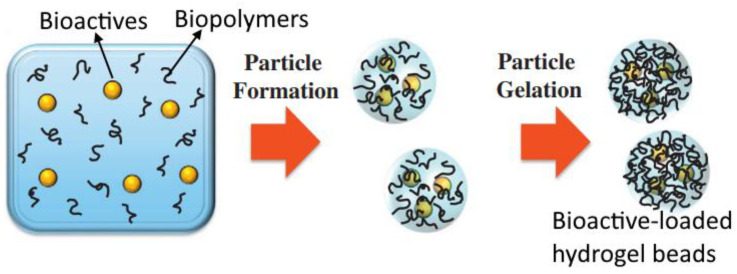
Illustration of a biopolymer-based delivery system for the encapsulation of bioactive compounds. Figure 6 is reprinted with permission from McClements [128] (Copyright © 2016 Elsevier B.V., Amsterdam, The Netherlands).

The addition of odor or bioactive compounds to a food formulation can improve its sensory, nutrient, and antibacterial properties [123]. Hydrogels are becoming increasingly popular as encapsulating agents, owing to their superior encapsulation efficiency, biocompatibility, low price, and ecofriendly characteristics [124]. Curcumin was encapsulated in hydrogels, with a 90.3% success rate [125]. CMC-K-car, like CMC gel, can encapsulate probiotic bacteria, but it does so at a much higher rate (94.7%) than CMC gel (74.7%). Therefore, it can be used to ensure the most effective release of bioactive substances [126]. The amount of curcumin released from LRA-CS (70%) was also reported to be lower than that from water (91%) in an in vitro small intestine model study [125]. To effectively administer lysozyme, a chitosan/sodium alginate hydrogel was developed in a recent study [124]. The relative activity of lysozyme was measured at 87.72%, but the rate at which bacteria were eliminated was as high as 100%. Figure 6 depicts the variety of hydrogel systems and the efficiencies with which they can encapsulate bioactive compounds. In another study, hydrogen was made with food-grade pectin and PEG (polyethylene glycol)-encapsulated Ca, vitamin D, Fe^2+^, and vitamin C. The gels were used to shield nutrients from artificial stomach acid [127]. Table 3 presents different types of hydrogel-based systems used for the encapsulation of bioactive compounds.

Bioactive substances provide specific health advantages to humans [134]. However, environmental stressors such as oxygen, temperature, and light can destroy these important molecules. In addition to external stress, the gastrointestinal tract imposes stressful conditions related to enzymes, pH, and other antinutrients in the meal [135,136]. Owing to the presence of dietary fiber, microcapsules have a high potential for target release [137]. As an oral delivery system, corn fiber gum-soy protein isolate hydrogel effectively encapsulates riboflavin. Adding corn fiber gum to pure soy protein isolate hydrogel was reported to enhance the riboflavin release efficiency in the gut [138]. In addition, the drug-carrying capacity alginate can be improved by enhancing its mechanical characteristics and durability [139]. Some researchers studied whether the microcrystalline and microfiber forms of cellulose contained in alginate beads may successfully enhance the mechanical characteristics and drug release of alginate beads in simulated intestinal fluid [140,141].

β-carotene is a bioactive molecule and vitamin A precursor with powerful antioxidant properties [142]. However, owing to its hydrophobic nature, direct application of β-carotene in food products is restricted. In addition, it is chemically unstable under environmental stresses such as oxygen, temperature, and light [143]. However, previous research has demonstrated that emulsion-filled hydrogels are a suitable vehicle for the targeted delivery of β-carotene [144]. In addition to emulsion-filled hydrogels that incorporate useful chemicals (such as beta-carotene) into the lipid phase, hydrogels can release entrapped oil into the intestinal environment by integrating certain biopolymers [144,145].

### 7.3. Delivery System

Hydrogels are often used in targeted delivery because they can absorb and store a large amount of water or biological fluids in a 3D network structure [146]. Nanoemulsions or Pickering emulsions have also been enclosed in hydrogel beads using Trojan horse nanoparticles [147]. Other hydrogel characteristics including stimulus reactivity to pH, heat, and light are especially helpful for regulated release in food nutrition delivery methods [148].

It should be noted that many studies have been conducted using hydrogels to create smart drug delivery systems, such as microgels. The majority of the time, hydrogels for controlled release medication administration is created based on the swelling or shrinkage caused by pH and temperature signals [149]. There are parallels between the food supply chain and the pharmaceutical supply chain, but there are also some distinctions between them [149]. Therefore, previous studies of the implementation of structured hydrogels with well-designed drug delivery systems serve as instructive examples [149].

### 7.4. Calorie Control

Reduced-fat or reduced-starch goods must be created immediately. By increasing satiety or decreasing consumption, hydrogels can also support weight loss and calorie restriction. Hydrogel particles made of protein and dietary fiber have a pleasant texture and can serve as a healthier alternative to starch granules [150]. The caloric density of pancakes that were cooked at temperatures significantly higher than the boiling point of water was reported to be reduced using a temperature-insensitive, food-grade, mixed agar–methylcellulose hydrogel [151]. Furthermore, vegetable oils can be used in place of animal oils to create emulsion hydrogels for use in low-fat meals.

### 7.5. Food Texture Perception

In the food engineering and processing field, one of the most important elements affecting food product quality is the impression of food texture [152]. Hydrogels are soft substances with important textural qualities, such as elasticity, hardness, chewiness, fracture, etc. [153]. Therefore, hydrogels can be utilized to enhance the mouth feel and texture of food, supporting low-calorie consumption [154]. For instance, emulsion hydrogels can affect the texture of food. It is interesting to note a reduction in the amount of calories in food can also be accomplished by replacing meat or grain with hydrogels with excellent textural properties or containing little oil [155].

### 7.6. Risk Monitoring

Hydrogels have attracted considerable interest in the food industry, as they can be used as a biosensor or signal probe to identify danger or risk elements in food. For instance, a fluorescent DNA hydrogel aptasensor was developed for sensitive ochratoxin A detection. A portable pH meter with an aptamer-responsive, crosslinked hydrogel was also proposed to detect aflatoxin B-1. In a related study, researchers produced a 2D molecularly imprinted photonic crystal hydrogel sensor for detection of oxytetracycline in milk [156]. In recent decades, polymer-based hydrogels and solid-phase extraction have been used as a sampling technique for direct onsite rapid detection of dangerous chemicals such as rhodamine B, mercury (II), and (Hg^2+^) ions [157].

### 7.7. Food Packaging Materials

The widespread use and abuse of high-molecular-weight, petroleum-based polymeric materials is a severe problem that must be addressed, and packaging food using hydrogel is a significant step in that direction [158]. Biobased biodegradable materials have been promoted for food packaging by recent research and scientific advancements [159]. The primary function of these components in food packaging is to regulate the humidity inside the container. Hydrogels can be used to reduce water activity (a_w_), preventing the growth of mold, microbes, and pathogens that cause spoilage of packaged foods and hygroscopic products. Hydrogel can also protect dry, crunchy items from becoming softer [160,161]. Mechanical characteristics, high absorption, water-absorbing properties, and even breathability and accountability are key properties to be concerned about from the standpoint of the formation of hydrogels. Moreover, traditional considerations such as minimal price, recyclability, and biocompatibility are essential features. Whether the hydrogels function as conventional, active, or smart packaging materials, the hydrogel structure is the most important factor to consider [162].

The headspace or the contained food product can interact directly with active packaging to prevent or inhibit microbial development, retaining the food’s nutritional value or enhancing its flavor over time [163]. Therefore, hydrogels have been thoroughly researched in food matrices and in vitro in combination with various antimicrobial chemicals, for example, silver or bioactive compounds [164]. Therefore, by integrating nanoparticles into the polymeric structure of a hydrogel, hybrid hydrogels such as nanocomposite hydrogels can represent a feasible solution to limitations such as the limited resistance to mechanical and hydrostatic stress of standard hydrogels [165]. Hydrogels also demonstrate their potential for use in intelligent packaging systems, whereby they can be used to alert consumers about the state of freshness of the food goods they contain or as a component of a quick test to identify pollutants [166]. The structured design of hydrogels is mostly used in this application area to support their smart response capabilities in the food engineering and processing field.

## 8. Conclusions and Future Directions

Gels are viscoelastic substances, and the commercial viability of food gels is of extreme significance. To develop gelled products, it is necessary to comprehend sol-gel transformation. One of the most crucial factors for consumers is the gel’s texture, which can be affected by a wide range of factors, such as the material’s makeup, composition, duration, temperature, pH, etc. The most recent developments in producing food-grade hydrogels for applications involving food were analyzed and discussed in detail in this study. In addition, aspects of hydrogels derived from natural sources were investigated; nevertheless, the gel strength of these substances could be improved by the addition of polysaccharides and proteins. Furthermore, hydrogels are a good source of energy, and when bioactive components are added, they act as a protective carrier for these substances. Owing to these attractive features, they are used in a wide range of food application areas, including for the production of bioactive substances, the protection of different flavors, and the replacement of fat in a wide range of products. In addition, consumers have demonstrated a high level of sensory acceptance for these hydrogel systems as fat replacers, sweetener replacers, etc., offering quality ingredients for a healthier lifestyle. Hydrogels can also be used in other contexts, such as for tissue regeneration, medical technology, and the precise administration of drugs.

Hydrogels have a wide range of uses in food processing, owing to their superior functional characteristics, consistency, mechanical strength, and water permeability. Nonetheless, it is still essential to pay attention to certain aspects of hydrogels. For example, it remains unknown how networks of hydrogels are interconnected. However, it is impossible to exert any control over the interaction of hydrogels. No accessible in-depth research has been conducted in this field to date. Regulation of the interplay between various gels can improve the absorption ability of hydrogels and cause less damage to the surrounding environment. As a result, further research and studies on this aspect of hydrogels are required.

## Figures and Tables

**Table 3:** Different types of hydrogel-based systems for the encapsulation of bioactive compounds.

Hydrogels	Form	Bioactive Compound	Encapsulation Efficiency (%)	Load Capacity	Ref.
Pectin-based	Core-shell hydrogel beads	Lactase	72	*	[129]
Xanthan gum	Biopolymer gels	Anthocyanin	*	*	[130]
Gelatin and chitosan	Gelation	Epigallocatechin gallate	95	*	[131]
Alginate-based nanohydrogel	Nanohydrogel	DOX (anticancer drug)	90	35%	[132]
Rice-protein varnish	Nanocomposite	Apigenin	91	92.5 mg·g^−1^	[133]

*: Not reported.

## Data Availability

Data are available within the article.
